# Biomarkers and Breakdowns: Neuroinflammatory Drivers Linking Sleep Disorders and Chronic Pain

**DOI:** 10.3390/biomedicines14010116

**Published:** 2026-01-06

**Authors:** Bento Alves, Isaura Tavares, Daniel Humberto Pozza

**Affiliations:** 1 Unit of Experimental Biology, Department of Biomedicine, Faculty of Medicine, University of Porto, 4200-319 Porto, Portugal; bentoeoa@gmail.com (B.A.);; 2 Institute for Research and Innovation in Health and IBMC, University of Porto, 4200-135 Porto, Portugal

**Keywords:** chronic pain, sleep–wake disorders, inflammation mediators, cytokines, neurodegenerative diseases, melatonin, opioid analgesics, metabolic diseases, pharmacological phenomena, bidirectional communication

## Abstract

Chronic pain and sleep disturbances are frequently associated and profoundly affect the quality of life, creating intertwined physical, emotional, and social challenges. This narrative review synthesizes current evidence on the molecular mechanisms and pharmacological influences underlying this bidirectional relationship. Elevated pro-inflammatory cytokines (IL-1β, IL-6, IL-10, TNF-α), neurodegenerative markers (tau, β-amyloid 42), metabolic hormones, and fasting glucose have been consistently associated with both objective and subjective sleep impairments in chronic pain conditions. Pharmacological agents such as melatonin and opioids exhibit heterogeneous effects on neurophysiological pathways, reflecting differences in mechanisms of action and their modulation of biological processes. Rather than offering therapeutic recommendations, this review aims to clarify how these mediators and drugs shape the complex interplay between pain and sleep. Overall, the evidence suggests that persistent dysregulation of inflammatory, neurodegenerative, and metabolic pathways may drive the reciprocal and detrimental interaction between chronic pain and sleep disturbances, highlighting opportunities for targeted research and integrated clinical strategies.

## 1. Introduction

Chronic pain (CP) persisting beyond three months can manifest either as a disease (“chronic primary pain”) or as a symptom secondary to another condition (“chronic secondary pain”) [1,2]. CP affects around 20–30% of adults worldwide and is the leading cause of years lived with disability. Its annual economic burden, estimated at $560–635 billion in the U.S. and accounting for 1.5–3% of gross domestic product in Europe, exceeds the combined costs of heart disease, cancer, and diabetes [3,4]. CP is increasingly recognized as a multifactorial disorder driven by complex neurobiological changes rather than simply prolonged nociception. It is sustained by both peripheral and central sensitization, along with maladaptive plasticity in pain-related neural networks. Peripheral sensitization arises from tissue injury or infection, where immune cell infiltration and activation of resident cells release inflammatory mediators such as cytokines, prostaglandins, ATP, and nerve growth factor. Nociceptors also respond to pathogens, danger signals, and miRNAs, while releasing neuropeptides like substance P and CGRP to promote neurogenic inflammation, heightening pain sensitivity [5,6,7]. Central sensitization, which can lead to chronic pain, arises when hyperactive primary sensory neurons release neurotransmitters and neuromodulators such as glutamate, substance P, CGRP, and BDNF in the spinal cord. Effects in the brain should also be considered in central sensitization, namely due to increased ascending transmission from the spinal cord. Overall central sensitization may lead to increased pain sensitivity both from the initial injury site and adjacent regions. While acute inflammation produces transient central sensitization, CP is associated with persistent neuroinflammation and long-lasting sensitization, mediated by mechanisms including NMDA receptor activation, MAPK signaling, and TLR4, along with involvement of glial cells, namely microglia and astrocytes [6,8,9,10,11].

Pain has traditionally been classified as nociceptive or neuropathic, yet many chronic pain conditions, such as migraine, fibromyalgia, and complex regional pain syndrome, persist without clear evidence of tissue damage, leading to the introduction of the term “nociplastic pain” as a mechanism driven by altered nociception [12]. Rather than a diagnosis, nociplastic pain reflects dysfunctional pain processing and central sensitization within brain circuits, often presenting with widespread pain, fatigue, sleep problems, sensory hypersensitivity, cognitive dysfunction, anxiety, and depression. Although proposed to arise autonomously, evidence indicates that persistent central sensitization usually requires ongoing noxious input or subtle, currently undetectable pathology, suggesting that these maladaptive neural changes may be rooted in occult or underrecognized tissue or neural abnormalities. Critically, nociceptive, neuropathic, and nociplastic mechanisms frequently coexist within the same individual, contributing to the complexity and treatment resistance of chronic pain. In this context, nociplastic pain should be understood as a neural mechanism tied to altered neurotransmission and circuit dysfunction rather than used as a stand-alone diagnostic label [13,14,15].

Additionally, altered interactions between neurons and glial cells contribute to heightened production of pro-inflammatory cytokines, amplifying nociceptive signaling and maintaining pain hypersensitivity. These processes not only intensify pain perception but also lead to distinct structural and functional alterations within the brain, underscoring chronic pain as a condition involving widespread neuroimmune dysregulation [8,16,17,18].

Similarly, sleep disturbances are linked to higher levels of biomarkers such as C Reactive protein (CRP), cortisol, tau, β-amyloid 42, and fasting glucose, and these alterations can affect both CP and sleep, reflecting a shared biological signature [11,19,20,21,22]. Sleep disturbances are highly prevalent among individuals with CP, affecting sleep quantity, quality, and circadian timing. It is suggested that CP and sleep problems frequently coexist due to shared pathophysiological mechanisms, including dysregulated inflammation, altered neurotransmission, and disrupted bidirectional pain–sleep modulation [23,24,25,26]. Sustained nociceptive input can induce neuroplastic changes that diminish the inhibitory effects of the locus coeruleus–norepinephrine system, facilitating the development of CP and related comorbidities such as anxiety, depression, and sleep disturbances [27,28,29]. On the other hand, elective activation of the locus coeruleus–spinal cord noradrenergic pathway alleviates neuropathic pain by enhancing norepinephrine release in the spinal dorsal horn, inhibiting microglial and astrocyte activation, and shifting local cytokine expression toward an anti-inflammatory profile [29]. Sleep loss further exacerbates these processes, as local changes in glial activity and neuroimmune signaling have been observed following sleep deprivation. Notably, such alterations may occur primarily within neural tissue and may not be detectable through peripheral blood measures, highlighting the importance of central mechanisms in the relationship between sleep and CP [30,31]. Experimental studies indicate that interleukin-1 (IL1) receptor 1 on neurons and astrocytes differentially regulates sleep patterns, highlighting the role of central IL-1 signaling in modulating sleep homeostasis under both physiological and stress conditions [32].

Sleep disorders are clinically defined conditions that meet specific diagnostic criteria, such as insomnia and restless legs syndrome (RLS) [33,34,35,36,37]. Poor sleep quality affects up to three-quarters of individuals with chronic pain, amplifying pain perception and contributing to the persistence of symptoms [38,39]. Although insomnia appears to predispose individuals to chronic pain and to exacerbate existing painful conditions, the temporal and causal dynamics remain unclear [40,41]. Prospective data suggest that baseline sleep problems increase both short- and long-term risk of chronic musculoskeletal pain, although evidence for specific sleep problems categories is uncertain [41]. Baseline chronic pain can disrupt sleep, making it hard to fall or stay asleep, reducing quality, and altering sleep architecture, and may increase short-term sleep disturbances. However, long-term effects remain uncertain [41,42,43]. Conversely, short, or disturbed sleep lowers pain thresholds and heightens pain intensity, creating a vicious cycle that further impairs sleep [44,45,46]. Together, these factors negatively affect both physical and psychological functions, affecting the overall quality of life [47,48]. The bidirectional relationship between CP and sleep involves multiple neurophysiological systems, including the opioid, monoaminergic, orexinergic, and immune systems, as well as the HPA axis and signaling molecules like melatonin, adenosine, and nitric oxide [49,50,51,52]. Notably, descending norepinephrine modulation from the brain, namely from the locus Coeruleus, modulates pain perception by inhibiting nociceptive transmission at the spinal cord [27,29,53].

Conservative, non-pharmacological interventions are universally recommended as first-line treatments for insomnia [54,55]. Guidelines recommend cognitive–behavioral therapy (CBT) for insomnia as the cornerstone of management, with any tapering of sleep medications integrated into care [56,57,58,59]. Pharmacotherapy is reserved for short-term use due to the limited sustained benefit, common adverse effects, and risk of dependence [59,60]. Additionally, CBT is a promising non-pharmacological intervention for patients with comorbid insomnia and CP [61,62]. Nevertheless, because this narrative review aimed to identify molecules influencing the relationship between chronic pain and sleep, we also incorporated studies of pharmacological agents and their effects on sleep, as these dysregulated pathways may represent potential targets for therapeutic intervention [51,63,64,65,66,67,68,69,70,71].

It is important to emphasize that optimal chronic pain management should follow the biopsychosocial model, integrating biological, psychological, and social interventions within a multidisciplinary framework aimed at reducing or eliminating the need for pharmacological treatments [72,73]. In the recommended multimodal approach to CP management, pharmacological treatments are one component alongside physical, psychological, and other non-pharmacological interventions. When medication is indicated, options may include opioid analgesics, tricyclic antidepressants, serotonin–norepinephrine reuptake inhibitors, and antiepileptics, although these agents carry risks such as adverse effects and potential dependence [74,75,76,77,78].

This narrative review aimed to synthesize evidence on the molecular and neurobiological mechanisms in sleep problems and chronic pain in humans, including the role of pharmacological agents in modulating the involved pathways. Unlike previous reviews focused on clinical management [41,79], the emphasis here is on mechanistic interactions rather than therapeutic recommendations.

## 2. Search for Existing Evidence in Human Studies

To summarize the existing evidence, comprehensive literature searches were conducted in Web of Science, PubMed, and Scopus databases in March of 2025. The search strategies were adapted to the syntax of each database, using the following keywords: chronic pain, sleep–wake disorders. Detailed information is described in Appendix A (Table A1).

The inclusion criteria comprised English-language articles involving humans with chronic pain, defined as pain persisting or recurring for longer than three months [2], that reported on pharmacological interventions or biological mediator measurements, including measurements of biological processes related to the cause of disease pathophysiology (e.g., pro-inflammatory cytokines, neurodegenerative markers, metabolic hormones), and that evaluated at least one sleep-related condition (e.g., insomnia symptoms or disorder, restless legs syndrome, sleep-disordered breathing, or sleep paralysis) or a measurable sleep outcome such as perceived sleep quality or daily sleep interference.

Chronic oncologic pain was excluded because pain and sleep disturbances in cancer populations are strongly influenced by disease-specific factors (e.g., tumor progression, chemotherapy, radiotherapy, immune suppression, cachexia, and psychological burden), which introduce multiple confounders. These mechanisms differ substantially from non-malignant chronic pain and would limit the comparability of biological markers and pharmacological effects [80,81,82,83]. Cannabis and alcohol were also excluded due to their complex, bidirectional effects on sleep and pain, as both substances can produce short-term analgesic or sedative effects but may worsen sleep architecture, pain sensitivity, and dependence-related hyperalgesia over time. Their impact is further complicated by variability in dose, route of administration, tolerance, and recreational versus medical use (cannabis), making it difficult to evaluate them alongside standardized pharmacological treatments. Importantly, cannabis and alcohol are also not widely or consistently prescribed for chronic pain and sleep disorders on a global scale, which reduces their relevance to a review of commonly used therapeutic agents [84,85,86,87,88].

Only human studies were included to ensure clinical relevance and to focus on biological markers and treatment effects directly applicable to individuals experiencing chronic pain with sleep disturbances. Although animal models and cell-culture studies provide valuable mechanistic insight, their experimental conditions often do not reflect the complexity of chronic pain, comorbid sleep disorders, psychological influences, or multimorbid states observed in humans [89,90,91]. Similarly, review articles were excluded to avoid overlapping information, minimize secondary interpretations, and prevent bias introduced by synthesized or selectively summarized data. Limiting the analysis to primary human research allows for a more direct and reliable examination of biologically measured markers and therapeutic outcomes in real-world clinical populations.

The 33 selected studies comprised 10 to 12,348 participants, detailed information is described in Appendix A (Table A2 and Table A3). Seven studies were limited to women [21,92,93,94,95,96,97] and one did not provide information on sex [98]. Most studies focused on unspecified chronic pain or included multiples etiologies, while some targeted specific diagnoses, with the most common being fibromyalgia and chronic back pain. Regarding sleep disorders, the most common was insomnia followed by sleep apnea. A comparable number of studies did not focus on a specific sleep problem but instead analyzed the perceived sleep quality of the patients.

Inflammatory cytokines (IL-1β, IL-6, IL-10, and TNF-α) were elevated in CP patients with a positive association with sleep problems [95,96,97] (Appendix A Table A1). Cortisol and fasting glucose were found to be increased in chronic widespread pain but had no impact on RLS severity [21]. Serum tau and β-amyloid 42 were increased in fibromyalgia patients, with both showing a positive association with PSQI scores [22]. Biological aging, measured by DNA methylation-based epigenetic clocks, was found to be accelerated in patients with high-impact CP with increased insomnia severity and decrease functional performance [99].

An association between opioid use and sleep problems/CP was observed [100,101,102,103,104]. Sleep problems tended to worsen with opioid use [66,103,105], which can increase wake after sleep onset (WASO) [102]. However, sleep improved with pregabalin [106], which reduced WASO and the number of awakenings, and also with certain opioids [104,105,107]. Suvorexant helped maintain sleep quality by reducing WASO [94], and melatonin also contributed to lower WASO [108]. Trazodone improved sleep in patients with somatoform pain disorder and insomnia by reducing awakenings and increasing deep sleep and oxygen saturation [109]. Suvorexant further reduced WASO and the duration of awakenings, though not the number of awakenings [94].

Number of hours of sleep or total sleep period (TSP) increased in two studies [102,104] on opioids but decreased in one study on trazodone [109]. Total sleep time (TST) increased in four studies, each involving a different drug: morphine [104], pregabalin [106], clonidine [110] and suvorexant [94]. Besides increasing sleep problems, opioids can also worsen physical and emotional symptoms [111,112,113]. Benzodiazepine use was associated with higher sleep problem index (SPI), while antidepressants and antipsychotics were linked to lower SPI [112].

**Figure 1:**
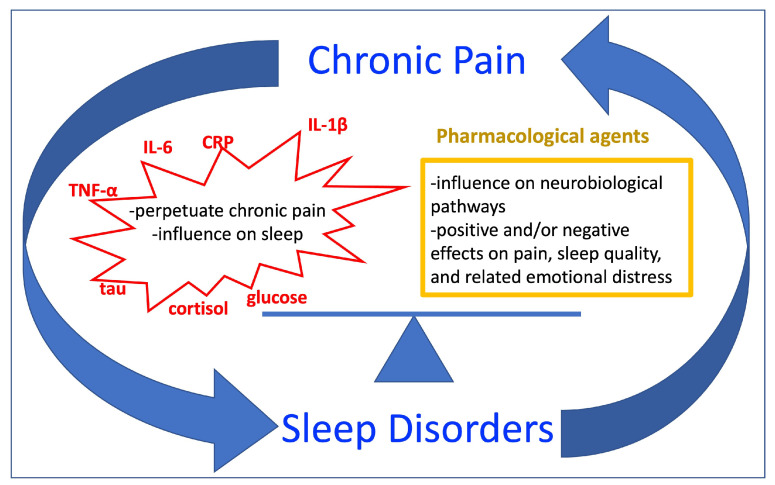
Graphical abstract of the main results. Legend: IL—Interleukin; CRP—C-reactive protein; TNF—Tumor necrosis factor.

Pain intensity can be reduced by opioids [104], amitriptyline [98], pregabalin [92,106], clonidine [110] and melatonin [93,108]; or increased with opioid use [105,113]. Both melatonin and suvorexant were found to reduce pain sensitivity [93,94]. A summary of the results is depicted in Figure 1.

## 3. Discussion

This manuscript synthesizes some of the current evidence on the effects of various biological mediators and the molecular influence of pharmacological agents on sleep and CP.

### 3.1. Biological Mediators

Inflammatory cytokines are frequently found increased in both CP and poor sleep quality, suggesting their potential role as a shared molecular pathway. Elevated levels of interleukin-6 (IL-6), commonly observed in patients with insomnia, are associated with heightened pain sensitivity, suggesting a shared inflammatory pathway underlying both conditions [20,96,97]. Moreover, patients with greater pain-related disability tend to exhibit poorer sleep quality and elevated cytokine levels, illustrating the co-occurrence of inflammation, sleep disturbance, and chronic pain severity [95]. These findings highlight inflammation as a potential therapeutic target in managing both sleep disruption and chronic pain.

Elevated levels of CRP may contribute to sleep problems in CP [19,95]. On the other hand, high-sensitive CRP (hsCRP) did not amplify the effects of insomnia and CP [101]. These differences might be influenced by the specific type and duration of chronic pain, as well as variations in the nature of the sleep problems and the characteristics of the studied populations. Therefore, while cytokines contribute to peripheral sensitization and central pain sensitivity [20,82], CRP shows inconsistent effects in CP and sleep problems. These inconsistencies highlight that CRP alone may not adequately reflect the low-grade, fluctuating inflammatory activity associated with chronic pain. In contrast, cytokine responses tend to be more dynamic and condition-specific, influencing neural pathways involved in pain modulation. This discrepancy emphasizes the complex and heterogeneous role of inflammatory mediators, which vary according to the underlying pain mechanism, the dominant type of sensitization (peripheral vs. central), comorbid sleep alterations, and differences in study design, including biomarker detection thresholds and sampling timing.

Metabolic, inflammatory, and neurodegenerative processes show overlapping features in CP patients with sleep problems. Elevated cortisol and fasting glucose levels have been observed in women with chronic widespread pain, caused by increased adrenergic sympathetic activity during sleep, indicating a potential link between CP and metabolic dysregulation [21]. Furthermore, elevated serum levels of tau and β-amyloid have been found in fibromyalgia patients, with a positive causative relationship between tau levels and sleep problems, suggesting that sleep problems may contribute to the neurodegeneration in fibromyalgia [22].

The shift from acute to CP appears to involve rapid DNA methylation reprogramming, highlighting its potential role in pain chronicity [114]. Furthermore, CP-conditioned states may exacerbate neurodegenerative processes by accelerating biological aging [115], measured via epigenetic clocks based on DNA methylation patterns, compared with pain-free controls.

Notably, higher epigenetic-aging scores are elevated in more severe insomnia symptoms and greater functional and activity limitations (lower quality of life). In patients with high-impact CP, these aging-related methylation changes also extend to key circadian clock genes, suggesting that epigenetic dysregulation of the molecular circadian machinery may further impair sleep–wake regulation and functional capacity [99,116].

### 3.2. Molecular Impact of Pharmacological Agents on CP and Sleep

This review also demonstrates that pharmacological agents exert molecular effects on the neurobiology of both sleep and pain. Although not considered first-line therapies, various drugs, including melatonin, opioid analgesics, antidepressants, and antiepileptics, have been frequently investigated and exhibit heterogeneous effects on both sleep and pain outcomes. While some drugs improved sleep efficiency and reduced pain intensity, others had mixed or negative effects on sleep quality and pain severity.

#### 3.2.1. Melatonin

Melatonin, an endogenous molecule widely recognized for its role in regulating circadian rhythms, can also be used as a pharmacological agent. Melatonin administration enhances sleep efficiency (SE) by modulating the suprachiasmatic nucleus of the hypothalamus, which controls the sleep–wake cycle. This modulation reduces WASO and increases total sleep time (TST), thereby improving sleep continuity. Melatonin also decreases sleep onset latency (SOL) by promoting the onset of sleep through its action on melatonin receptors MT1 and MT2, which are involved in the regulation of circadian rhythms [93,108].

CP patients often experience disrupted sleep patterns, which can lead to reduced melatonin levels. Additionally, melatonin has shown transient benefits in reducing pain in patients with severe CP conditions. This dual role is attributed to its anti-inflammatory and analgesic properties, which involve the inhibition of pro-inflammatory cytokines and modulation of pain pathways [108,117,118,119]. Melatonin improves sleep and alleviates pain in fibromyalgia and orofacial pain, demonstrating its potential to manage both in the same patient [93,108]. However, melatonin’s benefits are often transient, prompting the need for further evaluation of its long-term efficacy, dosing, and safety. Its effectiveness varies by individual and sleep problem cause, yet it remains a valuable option for sleep and pain management when combined with behavioral and lifestyle strategies [117,119,120]. Guidelines recommend exogenous melatonin for up to 3 months in patients with insomnia and over 54 years of age, particularly when cognitive–behavioral therapy (CBT) is not effective [59,60].

#### 3.2.2. Opioids

Despite the opioid crisis, particularly severe in the U.S., with its associated addiction and mortality, and despite opioids being primarily indicated for palliative care and acute pain, they remain a significant and well-studied option in chronic pain management [121,122,123]. Since our review aimed to examine the role of pharmacological agents in the interaction between chronic pain and sleep, we included opioids, not to advocate their use, but to impartially clarify their influence within this relationship. Pharmacological agents present a complex neurophysiological profile regarding their effects on sleep. Opioids can enhance sleep Stage 2 (S2) by modulating the activity of the central nervous system, but their effects on slow-wave sleep (SWS) are mixed, with some studies reporting increases and others decreases [63,68]. Opioids generally increase SOL by affecting the brain’s arousal systems but can reduce latency to persistent sleep (LPS) at lower doses, probably due to their sedative properties [104].

Opioids can improve SE by reducing awakenings and the arousal index, particularly in older adults. This is achieved through their action on opioid receptors, which modulate pain and stress responses, thereby promoting more stable sleep patterns [104,109]. However, chronic opioid use has been identified as a risk factor for central sleep apnea and ataxic breathing, with higher doses potentially exacerbating these conditions due to their depressive effects on respiratory centers in the brain [66,67,69].

The impact of opioids on sleep is further complicated by their dose-dependent effects. While low doses may improve certain sleep parameters, long-term, higher doses and opioid use disorder can lead to increased insomnia severity, sleep problems, fatigue, mental health problems, and respiratory complications [103,104,105,111,112,113,124,125,126,127]. Furthermore, opioids have been found to improve perceived sleep quality more than objective sleep outcomes in younger adults, with the opposite effect observed in older adults, mainly at higher doses [100,128]. These variabilities underscore the importance of individualized treatment plans and careful monitoring to balance the analgesic benefits with potential adverse effects on sleep and respiratory health [69,129,130,131]. It was demonstrated that baseline sleep problems negatively impact the effectiveness of pain treatments, and even opioids can be ineffective on CP [132]. Higher pain intensity worsens the opioid–insomnia link by reducing sleep quality and increasing wakefulness, while evening pain further exacerbates both sleep and opioid use [102,133]. These important nuances highlight the need for considering both subjective and objective measures when evaluating the effectiveness of opioid therapy on the bilateral relationship of pain and sleep.

#### 3.2.3. Other Pharmacological Agents

Although CBT is the first-line treatment for insomnia, pharmacological options, including benzodiazepines, benzodiazepine receptor agonists, orexin receptor antagonists, and low-dose sedating antidepressants, may be used for short-term management, with longer-term use considered in selected cases after careful evaluation of risks and benefits [59]. The bidirectional link between pain and sleep problems often involves emotional distress, which can be alleviated by antidepressants such as mirtazapine and trazodone. Notably, their effects are dose-dependent; at lower doses, particularly in the case of trazodone, the sedative properties are more prominent and are commonly used to address insomnia [71,98,109]. Mirtazapine increases slow-wave sleep (SWS) and reduces WASO, while trazodone enhances sleep continuity and reduces SOL, though it may cause next-day drowsiness. These effects improve sleep quality and mitigate the psychological distress of chronic pain, addressing both poor sleep and heightened pain sensitivity [71,98,109]. Additionally, certain antidepressants, such as selective serotonin reuptake inhibitors and serotonin–norepinephrine reuptake inhibitors, may decrease S2 sleep and promote respiratory problems [70,112,134]. These medications are particularly useful for patients who require both antidepressant and hypnotic effects. However, their use must be tailored to individual patient needs to minimize side effects and maximize therapeutic outcomes [135,136].

Among anticonvulsants, pregabalin reduces the release of neurotransmitters such as glutamate, norepinephrine, and substance P. This action decreases neuronal excitability and transmission of pain signals, making pregabalin effective in managing neuropathic pain [137]. Pregabalin has been shown to increase SWS and SE, reduce SOL and WASO, and enhance overall sleep quality [106]. These effects are particularly beneficial for patients experiencing both pain and sleep problems, providing a comprehensive therapeutic approach [104,109]. Anticonvulsants offer dual benefits for pain and sleep with fewer side effects, making them a preferred treatment for neuropathic pain.

Benzodiazepines, known for their anxiolytic and hypnotic effects, enhance GABA-A inhibition, reducing SOL and increasing TST for better sleep [110]. However, long-term use of benzodiazepines can lead to dependence and tolerance due to downregulation of GABA-A receptors and alterations in brain chemistry, necessitating careful management and consideration of alternative therapies for chronic use [138]. Antipsychotics are occasionally used off-label for sleep problems but may impair sleep quality and respiratory function, increasing sleep apnea risk [112]. Their use requires careful risk–benefit evaluation in CP patients.

Dual orexin receptor antagonists (DORAs), including suvorexant, lemborexant, daridorexant, and the recently approved ultra-short half-life agent vornorexant, offer a promising strategy for improving sleep in patients with CP. These agents inhibit both orexin receptors, OX1R and OX2R, which have distinct but complementary distributions and functions within the central nervous system. OX1R is implicated in arousal and nociceptive modulation, while OX2R plays a key role in stabilizing sleep–wake transitions and regulating REM sleep [139,140,141]. By blocking these receptors, DORAs reduce hyperarousal and may indirectly influence pain pathways, although evidence in humans remains under investigation [139]. Suvorexant has been shown to increase TST, reduce WASO, and lower pain sensitivity, although sedation and nausea are reported adverse effects [94]. Lemborexant similarly improves insomnia symptoms without significantly altering pain scores, with lightheadedness and daytime sleepiness among the most common side effects [142]. Daridorexant, characterized by a shorter half-life than suvorexant and lemborexant, offers comparable improvements in sleep onset and maintenance with a lower risk of next-day residual effects. Vornorexant, the newest DORA and the first with an ultra-short elimination half-life, expands pharmacologic flexibility by providing a rapid onset and shorter duration of action, potentially benefiting patients whose symptoms primarily involve difficulty initiating sleep [143]. Importantly, because DORAs affect REM sleep architecture, some patients may experience vivid dreams or nightmares [139,142,144]. Together, these findings underscore the putative rote of the orexin system in the interaction between sleep and CP and highlight the therapeutic potential of DORAs in this context, while acknowledging their limitations.

Understanding the site of action of pharmacological interventions is crucial for linking their effects to specific brain circuits involved in chronic pain and sleep regulation. Many analgesics and neuromodulatory drugs act on central pathways, modulating neurotransmitters such as glutamate, norepinephrine, and GABA, which influence both nociceptive processing and sleep–wake regulation. For example, the locus coeruleus–noradrenergic system, spinal and cortical glutamatergic circuits, and thalamocortical networks are implicated in pain perception as well as sleep homeostasis. Various drug classes target these pathways through different mechanisms: non-opioid analgesics inhibit prostaglandin production to reduce pain and inflammation; opioid analgesics act via opioid receptor inhibition; antidepressants modulate norepinephrine and serotonin signaling; benzodiazepines enhance GABAergic inhibition in the brain; anticonvulsants reduce glutamate and sensory neuropeptide release by decreasing calcium influx at synapses; local anesthetics block Na^+^ and K^+^ channels and regulate intracellular calcium; and corticosteroids provide anti-inflammatory effects that indirectly modulate nociceptive signaling. Collectively, these drugs illustrate the overlap between central neurotransmitter modulation, pain control, and sleep regulation [49,145,146,147,148]. By targeting these overlapping circuits, pharmacological treatments can simultaneously modulate pain sensitivity and sleep quality, highlighting the shared neurobiological substrates that underlie the frequent comorbidity of chronic pain and sleep disturbances.

### 3.3. Limitations

The division of results into biological mediators and drug effects highlights the heterogeneity of study designs and populations and may affect the generalizability of the findings. Seven studies were limited to women, potentially introducing sex bias, and most studies focused on unspecified CP or included multiple etiologies. While the true effect for adverse effects is likely close to the estimated effect, there remains a possibility of substantial or even significant differences for the other variables.

Cerebrospinal fluid (CSF) may offer more direct insight into the sleep–pain relationship, but its use is limited by the invasiveness of collection and risk of infection [149,150,151,152,153]; therefore, CSF biomarkers were not included in this review. Similarly, although large-scale genetic datasets could clarify shared mechanisms between sleep and chronic pain [154,155,156,157,158], they are beyond the scope of this review, which focuses on biological mediators and the molecular influence of pharmacological agents.

This manuscript does not aim to provide pharmacological recommendations for managing the complexity of CP and sleep problems. It is important to emphasize that the initial approach to treating sleep disturbances should be conservative, with pharmacotherapy reserved for carefully selected patients. Since this was not the primary focus of the manuscript, we did not investigate the best practice treatments for the complex relationship between CP and sleep problems. Instead, this study sought to identify the molecular factors that can influence and may be influenced by this intricate relationship, including emotional variables. Overall, the present review highlights potential associations between biological mediators, neural mechanisms, and the interplay of pain and sleep, offering a framework to guide future research while acknowledging that these links remain correlational and require further validation.

## 4. Conclusions

Multiple biological mediators appear to be associated with the co-occurrence of chronic pain and sleep disturbances and may be dependent on the severity of the condition. Elevated pro-inflammatory cytokines (IL-1β, IL-6, IL-10, TNF-α), neurodegenerative markers (tau, β-amyloid-42), metabolic hormones (cortisol), fasting glucose, and measures of accelerated epigenetic aging have all shown correlations with greater insomnia severity and disrupted sleep in conditions such as fibromyalgia and low-back pain. While these findings suggest potential shared biological pathways underlying pain and sleep problems, the current evidence is limited, largely observational, and cannot establish causality.

Taken together, these observations highlight important associations between biological mediators, pharmacological interventions, and the interplay of pain and sleep, emphasizing the complexity of their relationship. They also underscore the need for well-designed longitudinal and mechanistic studies to clarify causal links, identify reliable biomarkers, and optimize therapeutic strategies. Future research should particularly address unresolved conceptual questions surrounding nociplastic mechanisms, shared neurobiological pathways, and the influence of patient-specific factors, which may ultimately inform more individualized and effective approaches to managing chronic pain with comorbid sleep disturbances.

## Data Availability

No new data were created or analyzed in this study. Data sharing is not applicable to this article.

## Abbreviations

The following abbreviations are used in this manuscript:

CLBP	Chronic Low Back Pain
CP	Chronic pain
CRP	C-reactive protein
CBT	Cognitive–Behavioral Therapy
ESS	Epworth sleepiness scale
F	Female
IL	Interleukin
LPS	Latency to Persistent Sleep
M	Male
MOS-SS	Medical Outcomes Study-Sleep Scale
NAASO	Number of Awakenings After Sleep Onset
PVT	Psychomotor Vigilance Test
REM	Rapid Eye Movement Sleep
RLS	Restless legs syndrome
S2	Stage 2
SE	Sleep Efficiency
SOL	Sleep Onset Latency
SPI	Sleep Problems Index
SWS	Slow-Wave Sleep
TIB	Time in Bed
TNF	Tumor necrosis factor
TSP	Total Sleep Period
TST	Total Sleep Time
WASO	Wake After Sleep Onset

## Appendix A

**Table A1:** Detailed manuscript selection.

Phase	Number of Manuscripts	Reasons for Exclusion
Results using keywords	656 in Scopus, 512 in PubMed, 111 in Web of Science	272 manuscripts were removed due to duplication in the databases
Selection process	1007 screened by titles and abstracts	967 excluded as per the inclusion criteria
	40 selected	1 not retrieved
	39 comprehensively analyzed	6 excluded after full text analysis
Final inclusion	7 related to biological mediators (Table A2) and 26 to pharmacological agents (Table A3)	-

**Table A2:** Comparative overview of studies that search for molecules involved in chronic pain and sleep problems.

Ref.	Pain	Sleep	Molecule	Main Results
Ho et al., 2023 [101]	Chronic Low Back Pain	Insomnia	High-sensitive C-reactive protein	Risk of CP in patients with insomnia
Park & Chung, 2016 [95]	Temporomandibular Disorder	Perceived sleep quality	C-reactive protein, interleukins, tumor necrosis factor	Cytokines and interleukins elevated in sleep and temporomandibular disorders
Lerman et al., 2022 [97]	Temporomandibular Disorder	Insomnia	Interleukin-6	Higher pain severity, functional limitation, and interleukin 6 in insomnia
Hunt et al., 2022 [96]	Temporomandibular Disorder	Insomnia	Interleukin-6	Higher pain sensitivity and interleukin 6 in poor sleep
Stehlik et al., 2018 [21]	Chronic Widespread Pain	Restless Leg Syndrome, Sleep quality	Cortisol, Glucose, Ferritin	Higher sleep problems severity, cortisol, glucose, anxiety, and depression in CP
Thi Nguy et al., 2022 [22]	Fibromyalgia	Sleep quality	Tau, β-amyloid 42	Higher serum tau and β-amyloid 42 in poor sleep
Aroke et al., 2023 [99]	Chronic Low Back Pain	Insomnia	DNA methylation	Higher biological aging, insomnia severity, and reduced functional performance in high-impact pain.

Legend: CP, Chronic Pain.

**Table A3:** Comparative overview of studies evaluating the effect of drugs on chronic pain and sleep disturbances.

Ref.	Pain	Sleep	Treatments	Main Results	Adverse Effects
Rosenthal et al., 2007 [104]	Osteoarthritis pain ≥ 4	Perceived sleep quality	Morphine sulfate: 14 days 30 mg or 7 days 30 mg → 14 days 60 mg	Higher sleep duration, quality, and cognition with reduced pain after treatment	Nausea (*n* = 10) Sedation (*n* = 5) Constipation (*n* = 5) Vomiting (*n* = 4) Pruritus (*n* = 4) Sedation and unresponsiveness (*n* = 1)
Peles et al., 2009 [125]	Moderate to severe CP ≥6 months	Perceived sleep quality	High (>150 mg) or low (<80 mg) daily Methadone Maintenance treatment (≥3 months)	Higher sleep problems and awakenings with unchanged methadone levels	-
Jungquist et al., 2012 [66]	CP ≥ 6 months	Sleep Apnea	Morphine equivalent doses of 5–60 mg, 61–200 mg, or 201–960 mg	Higher sleep problems and pain with opioid use	-
Rose et al., 2014 [103]	CP	Sleep Disordered Breathing	Morphine 40–500 mg/day Oxycodone 30–350 mg/day Methadone 20–100 mg/day	Higher apnea severity, low arousal index, and impaired reaction time with opioid use	-
Morasco et al., 2014 [105]	Arthritis, Fibromyalgia, Low Back Pain, Migraine headache, Neck or Join Pain, Neuropathy (12.8 ± 11.5 years)	Perceived sleep quality	Opioids: average 34.6 ± 54.9 mg/day of Morphine equivalent	Higher sleep apnea, pain severity, and poor sleep quality with opioid use	-
Robertson et al., 2016 [127]	Chronic Back Pain (7.5 ± 8.6 years)	Perceived sleep quality	Opioids: high (~100 mg/day) or low (<100 mg/day) Morphine equivalent	Higher insomnia, fatigue, pain, and abnormal brain activity in CP with opioids	-
Yarlas et al., 2016 [107]	Chronic Low Back Pain (Moderate-severe pain for ≥12 weeks)	Perceived sleep quality	Buprenorphine 10/20 mcg/hour vs. placebo in opioid-naïve vs. Buprenorphine 20 mcg/hour vs. 5 mcg/hour in opioid-experienced	Higher sleep disturbance and lower pain severity associated with better sleep scores	-
Miller, Chan, Curtis et al., 2018 [102]	Pain ≥ 10/100 last 14 days	Insomnia	Opioids (14 days follow-up)	Lower sleep quality, and longer time in bed with opioid use	-
Curtis, Miller, Rathinakumar et al., 2019 [100]	Fibromyalgia (≥3 months)	Insomnia	Opioids (14 days follow-up)	Lower slow-wave sleep, and longer sleep onset with opioid use	-
Curtis, Miller, Boissoneault et al., 2019 [128]	Fibromyalgia (≥10/100 evening pain)	Insomnia	Opioids (14 days follow-up)	Higher subjective sleep improvements than objective measures with opioid use	-
Koffel et al., 2020 [132]	Chronic Back Pain and Osteoarthritis (moderate to severe pain almost daily for ≥6 months)	Perceived sleep quality	12 months opioid therapy vs. nonopioid therapy	Higher baseline sleep problems predicted less pain improvement	-
Ponce Martinez et al., 2020 [126]	Chronic non-specific pain (≥3 months)	Perceived sleep quality	Methadone (mean daily dose of 81 mg)	Higher sleep problems and pain catastrophizing associated with greater pain intensity	-
Miller et al., 2021 [133]	Fibromyalgia (≥3 months)	Insomnia	Opioids (average daily use of 1.75 ± 0.73 dosage units)	Lower sleep quality and higher evening opioid use with greater pain	-
Cody et al., 2022 [111]	Chronic non-specific pain (≥3 months)	Insomnia	Opioids	Greater insomnia severity in HIV with pain, especially with opioid use	-
Ellis et al., 2022 [124]	Chronic non-specific pain	Insomnia, Sleep Apnea, Sleep Paralysis, Restless Leg Syndrome	Opioids (use disorder treatment)	Sleep quality worsens in CP + insomnia; less decline in opioid use disorder	-
Wilson et al., 2023 [113]	Chronic Low Back Pain (≥4/10 pain for ≥3 months	Perceived sleep quality	Opioids: observational	Sleep disturbance linked to higher pain severity, pain interference, and reduced physical function; opioids strengthen these associations	-
Lintzeris et al., 2016 [112]	Chronic Back and Neck Pain, Arthritis or Rheumatism (most common)	Perceived sleep quality	Morphine equivalent 72.7 (36–145) mg/day Benzodiazepines 28.2 (25.6–30.8) Antipsychotics 7.2 (5.8–8.8) Antidepressants 54.4 (51.6–57.4) Antiepileptic 40.9 (38.1–43.8)	Long-term opioids and multiple pain conditions worsen sleep and respiratory issues; benzodiazepines increase, and some psych meds reduce sleep problems	-
Saletu et al., 2005 [109]	Somatoform Pain Disorder	Insomnia	Trazodone hydrochloride: 3 nights vs. control: 2 nights	Improved sleep with higher O_2_ saturation and reduced arousals	-
Calderon et al., 2011 [98]	Temporomandibular Disorder (≥moderate pain for ≥6 months, almost daily last month)	Perceived sleep quality	Amitriptyline 7 weeks: 25 mg, 25 mg + CBT, placebo + CBT or placebo	Pain improved (amitriptyline most), depression eased with CBT, sleep unchanged.	visual symptoms (*n* = 1)
Roth et al., 2012 [106]	Fibromyalgia	Sleep problem	Pregabalin 300–450 mg (4 weeks) → taper/washout (2 weeks) → placebo (4 weeks) or inverse	Better objective and subjective sleep with less pain and fatigue	dizziness (*n* = 32) somnolence (*n* = 23) headache (*n* = 8) nausea (*n* = 7)
Silverman et al., 2012 [92]	Abdominal Adhesion Pain (pain ≥ 4/10 for ≥3 months)	Daily sleep interference	Pregabalin 150/300 mg or placebo (8 weeks) → pregabalin 300 mg (4 weeks)	Reduced pain, no significant change in sleep interference	dizziness (*n* = 2) night sweats, headaches, hyperactivity, drowsiness, blurred vision, numbness (*n* = 1)
Bamgbade et al., 2022 [110]	Limb Pain (frequent and/or significant)	Insomnia	Zopiclone (3.75/7.5 mg) and Clonidine (0.1/0.2 mg) on alternate nights for 3 weeks	Clonidine improved pain and sleep; zopiclone caused more side effects	Zopiclone: confusion, amnesia, mood disorder, hallucination, locomotor dysfunction, nausea and headache Clonidine: dry mouth
Vidor et al., 2013 [93]	Temporomandibular Disorder (pain ≥ 3/10 for 7 days)	Perceived sleep quality	4 weeks Melatonin 5 mg or placebo	Pain and analgesic use decreased; pain threshold and sleep quality improved	-
Onyeakazi et al., 2024 [108]	CP ≥ 7/10 for ≥3 months	Perceived sleep quality	Melatonin 2 mg (6 weeks) → washout (2 weeks) → placebo (6 weeks) or inverse	Early improvements in sleep and pain, no long-term differences or adverse effect changes	7% reported side-effects
Roehrs et al., 2020 [94]	Fibromyalgia	Insomnia	Suvorexant 20 mg (9 nights) → washout (7 days) → placebo (9 nights)	More sleep, fewer awakenings, unchanged stages, and lower pain sensitivity.	suvorexant: residual sedation (*n* = 4) nausea (*n* = 3)
Ueno et al., 2024 [142]	Chronic non-specific pain	Insomnia	Lemborexant 5 mg (2 weeks) → Lemborexant 2,5/5/10 mg (2 weeks)	Reduced insomnia and no changes in pain	5 sleepiness or mood disorder

Legend: CP, Chronic Pain; CBT, cognitive–behavioral therapy; *n*, number of reports; -, information not available.
